# Pan-cancer analysis and experimental validation of FPR3 as a prognostic and immune infiltration-related biomarker for glioma

**DOI:** 10.3389/fgene.2024.1466617

**Published:** 2024-10-09

**Authors:** Chenglin Ye, Peng Li, Boxu Chen, Yong Mo, Qianrong Huang, Qiuyun Li, Qinhan Hou, Ligen Mo, Jun Yan

**Affiliations:** ^1^ Department of Neurosurgery, Guangxi Medical University Cancer Hospital, Nanning, China; ^2^ Department of Neurosurgery, Institute of Brain Diseases, Nanfang Hospital of Southern Medical University, Guangzhou, China; ^3^ Department of Breast Surgery, Guangxi Medical University Cancer Hospital, Nanning, China

**Keywords:** FPR3, pan-cancer, glioma, biomarker, immune infiltration

## Abstract

Formyl peptide receptor 3 (FPR3) is known to have implications in the progression of various cancer types. Despite this, its biological significance within pan-cancer datasets has yet to be investigated. In this investigation, we scrutinized FPR3’s expression profiles, genetic alterations, prognostic significance, immune-related characteristics, methylation status, tumor mutation burden (TMB), and microsatellite instability (MSI) across different types of cancer. We utilized TISCH’s single-cell data to identify immune cells closely associated with FPR3. The predictive significance of FPR3 was evaluated independently in gliomas using data from TCGA and CGGA datasets, leading to the development of a prognostic nomogram. Immunohistochemistry and Western blot analysis confirmed FPR3 expression in gliomas. Lastly, the CCK-8 and wound-healing assays were employed to assess the impact of FPR3 on the proliferation and metastasis of GBM cell lines. In numerous cancer types, heightened FPR3 expression correlated with adverse outcomes, immune cell infiltration, immune checkpoints, TMB, and MSI. In glioma, FPR3 emerged as a notable risk factor, with the prognostic model effectively forecasting patient results. The potential biological relevance of FPR3 was confirmed in glioma, and it was shown to have significant involvement in the processes of glioma growth, immune infiltration, and metastasis. Our results imply a potential association of FPR3 with tumor immunity, indicating its viability as a prognostic indicator in glioma.

## 1 Introduction

Cancer is a significant public health challenge on a global scale and is a primary cause of mortality. The 2020 report from the World Health Organization indicated that cancer was responsible for approximately 10 million global fatalities, with Asia accounting for 58.3% of these fatalities (Sung et al., 2021). The survival rates of numerous malignancies are dismal, despite scientific endeavors to enhance and create novel treatments. Presently, targeted therapy is bringing about a fundamental change in cancer treatment approaches (Trajanoska et al., 2023). Pan-cancer analysis,a method that aims to identify the shared and unique characteristics of a variety of malignancies (Weinstein et al., 2013), has been demonstrated to be a valuable instrument for investigating the genetic and molecular underpinnings of various cancer types (Ju et al., 2021).

FPR3, also referred to as FPRL2, is a G protein-coupled receptor. While FPR1 and FPR2 have been extensively studied, research on FPR3 is relatively limited. Studies have shown that FPRs facilitate cancer progression by promoting cell proliferation and metastasis (Cheng et al., 2014; Li et al., 2017). Astrocytes and microglia expressing FPRs have essential roles in pathogen detection and initiating the inflammatory response (Shastri et al., 2013). In their constant pursuit of expansion, tumors have effectively used FPRs. Highly malignant human glioma cells extensively express FPR1, accelerating this aggressive illness (Huang et al., 2010). The endogenous ligand ANXA1, abundant in deceased tumor cells, enhances immune response and the migration of glioblastoma cells through the FPR1 pathway (Zhou et al., 2005). Additionally, human astrocytoma cells upregulate FPR1 (Boer et al., 2013). Human brain tumors, known for their poor survival rates, also exhibit FPR1 (Snapkov et al., 2016). Thus, FPR overexpression in neoplastic epithelial cells promotes tumor development, invasion, and metastasis. Compared to FPR1, research on FPR3 is significantly less extensive. Individual survival has been associated with FPR3 expression, which is considerably increased in breast cancer tissue relative to healthy tissue (Qi et al., 2020).

Although FPR3 has been examined in specific malignancies, it remains excluded from extensive cancer analyses. This research aimed to evaluate the prognostic impact, genetic alterations, and FPR3 expression across various cancer types. Subsequently, we explored the connections between FPR3 and immunomodulators, TMB, and MSI. Furthermore, we analyzed single-cell associations between FPR3 and immune cells. Data from The Cancer Genome Atlas (TCGA) and the Chinese Glioma Genome Atlas (CGGA) were utilized to conduct clinical correlation analysis, independent prognostic evaluation, creation of a nomogram, and biological function assessment in glioma cases.

## 2 Materials and methods

### 2.1 Differential analysis and prognosis evaluation in pan-cancer

Sanger Box was an online database used to analyze FPR3 expression across cancers. Expression data for the FPR3 gene were obtained from individual cancer samples. The analysis of the associations between FPR3 levels and overall survival (OS), progression-free interval (PFI), and disease-specific survival (DSS) in cancer patients was conducted using the Sanger Box tool.

### 2.2 Immunomodulator analysis and single-cell analysis

The examination of the relationships between FPR3 and immune checkpoint elements, encompassing inhibitory and stimulatory factors, along with major histocompatibility complexes (MHC), was carried out using the TISIDB database. Recent research has focused on predictive biomarkers such as high TMB and high MSI to evaluate immunotherapy efficacy (Chan et al., 2019). Spearman’s rank assessed the association of FPR3 expression with TMB or MSI, followed by the utilization of the R “fmsb” package to produce a radar chart illustrating these results. Furthermore, UMAP plots depicting the expression profiles of FPR3 across various cell types were acquired from the TISCH database (Sun et al., 2021).

### 2.3 Genomic alterations of FPR3 across cancers

This research also investigated the genomic alterations in FPR3 using the cBioPortal database. Additionally, we employed GSCA (Liu et al., 2018) to examine the copy number variation (CNV) of FPR3. The analysis of the associations between FPR3 levels and overall survival (OS), progression-free interval (PFI), and disease-specific survival (DSS) in cancer patients was conducted using the Sanger Box tool.

### 2.4 Data source and processing of glioma cohort

The present study focused on four distinct cohorts comprising individuals diagnosed with gliomas. Data on gene expression and pertinent clinical details were sourced from reliable databases, including the TCGA and CGGA databases (Zhao et al., 2021), as well as the Rembrandt (Gusev et al., 2018) and Gravendeel databases (Gravendeel et al., 2009), accessed through their respective online portals. The Rembrandt and Gravendeel databases were accessed via the Gliovis website (Bowman et al., 2017). The TCGA dataset served as our primary resource for investigating the prognostic implications of FPR3 in glioma. To ensure the validity and robustness of our findings, we further utilized three additional cohorts to validate and reinforce the results.

### 2.5 Clinicopathological correlation and prognostic analysis of FPR3 in glioma

The Wilcoxon test was used to examine the levels of FPR3 and its corresponding clinical features. Variables such as age, sex, histologic grade, isocitrate dehydrogenase (IDH) mutation status, 1p19q codeletion status, and MGMT promoter (MGMTp) methylation status were evaluated. The R package “ComplexHeatmap” was employed to create a heatmap that compared the distribution of clinical features across various FPR3 expression groups. Box plots illustrated the variations in FPR3 expression among different clinical categories. The “Survminer” package in R was utilized to build the Cox model and perform sequential variable selection. These findings were validated using the CGGA cohort.

### 2.6 The process of formulating and scrutinizing a nomogram

The nomogram is a widely employed tool in oncology, providing personalized prognostic information for cancer patients. It offers easily understandable risk assessments (Iasonos et al., 2008). A representative nomogram model was developed using the “RMS” package in R. This model integrated the FPR3 transcription level with traditional clinical factors such as grade, age, and IDH status. Each patient’s composite score was transformed by the nomogram into the predicted probability of outcome duration. The effectiveness and predictive capability of the nomogram were evaluated using statistical methods, including the concordance index (C-index), time-dependent receiver operating characteristic (tROC) curve, calibration curve, and decision curve analysis (DCA).

### 2.7 Scanning for DEGs and enrichment analysis of function

The median value was used to dichotomize FPR3 expression, creating a categorical dependent variable. Differentially expressed genes (DEGs) in the TCGA dataset were pinpointed through the Wilcoxon rank test, applying screening criteria of a false discovery rate (FDR) < 0.05 and |log fold change (logFC)| > 1. To ensure accuracy, a minimum average expression level threshold of 1 for the raw DEGs was established. DEGs that met the specified criteria were chosen for additional analysis. Visualizations were generated using R, with a volcano plot illustrating all DEGs and a heatmap depicting the expression profiles of the top 50 DEGs. The Gene Ontology (GO) and Kyoto Encyclopedia of Genes and Genomes (KEGG) tools were utilized, and the results were displayed with bubble charts. Discrepancies between two biological states were assessed for statistical significance using Gene Set Enrichment Analysis (GSEA). with each analysis involving 1,000 gene set permutations. FPR3 levels were used as a distinguishing phenotype indicator. Enriched networks for each phenotype were evaluated based on their minimal *p*-value.

### 2.8 Analysis of immunomodulators related to the FPR3 receptor

We measured FPR3 expression in 28 TIL categories using the Tumor Immunology and Signaling Database (TISIDB) (Ru et al., 2019) to investigate FPR3-associated immune infiltration. The TISIDB database integrates diverse data sources pertinent to tumor immunology. FPR3 and TILs were examined using Spearman correlation analysis. TISIDB was implemented to analyze FPR3 levels in LGG immune subgroups. FPR3 expression was predominantly detected in three distinct subtypes, namely C3 (characterized by inflammation), C4 (characterized by lymphocyte depletion), and C5 (characterized by immunological quiescence). We computed stromal, immune, and ESTIMATE scores for each sample utilizing the ESTIMATE (Estimation of Stromal and Immune Cells in Malignant Tumor Tissues Using Expression) methodology. Furthermore, the proportions of 22 tumor immune cell types were assessed using CIBERSORTx. Subsequently, the TCGA dataset was analyzed with the R package “ggplot2” to investigate the relationship between FPR3 and immune checkpoints. GEPIA2 also identified statistically significant relationships between immune cell gene markers and FPR3 expression.

### 2.9 Correlation of FPR3 with drug sensitivity

Glioma-related RNA sequences were obtained from the TCGA dataset. Subsequently, the IC50 values of widely used chemotherapeutic drugs were computed using the R package “oncoPredict,” which predicts chemotherapy response by leveraging tumor gene expression profiles. These IC50 values were then compared across subgroups with high and low FPR3 expression to determine therapy efficacy.

### 2.10 Tissue samples collection

The Department of Neurosurgery supplied glioma and non-tumor specimens spanning the period from 2020 to 2023. Following the surgical removal of the tissue, the samples were promptly frozen at a temperature of −80°C and then transferred to a container containing liquid nitrogen for extended preservation. Surgically resected tumor tissue specimens were sectioned and processed in the pathology department. Histological features were assessed using light microscopy. Immunohistochemical staining was performed to detect specific markers, such as IDH mutations and ATRX deletions. These analyses were essential for confirming the diagnosis and ensuring that the tumor grading adhered to the WHO CNS5 diagnostic criteria. Out of the 63 samples initially obtained, 41 samples with inadequate information were eliminated, leaving 22 samples with a clear diagnosis for further study.

### 2.11 Immunohistochemistry

Initially, the tissue slices underwent dewaxing and subsequent hydration through a series of ethanol solutions of increasing concentrations. This process was followed by antigen retrieval using a sodium citrate solution. In order to suppress endogenous peroxidase activity, a 30% hydrogen peroxide and methanol solution was employed. The transparencies were subsequently incubated with a primary antibody solution overnight (anti-FPR3, orb39488, Biorbyt, Cambridge, United Kingdom). Staining was performed using the DAB kit from Beyotime, China, and hematoxylin. Visual examination of the slides was conducted using a Leica laboratory microscope manufactured in Germany.

### 2.12 Cell line and transfection

U251 human glioblastoma cell lines were obtained from the cell bank of the Chinese Academy of Sciences (Shanghai, China). In DMEM medium with 10% foetal bovine serum (Gibco, China) at 37°C and 5% CO_2_, U251 cells were cultivated daily. All experiments used logarithmic-growing cells. Sangon Bioengineering (Shanghai, China) chemically synthesised FPR3 siRNA. Lipofectamine 3000 from Invitrogen was used to transfect FPR3 siRNA and negative control (NC) cells as specified. The following siRNA sequence were used in this study:

FPR3-siRNA: 5′-AAC AAC AUC UCU UUG AGC C-3′

### 2.13 CCK-8 assays

U251 cells were divided into two groups: NC group and Si-FPR3 group. After 24 h of transfection, trypsin digestion was resuspended and counted by cell counter, and 96-well plates were inoculated with 1,000 cells per well, and 5 replicate wells were repeated. After incubation for 1.5 h in the absence of light, the plates were set at 37°C and OD450 quantification was performed.

### 2.14 Wounding healing assays

U251 cells were inoculated into six-well plates 200,000 cells per well and incubated overnight in cell culture incubator, waiting for them to grow to 80–90 confluence, lipo3000 was transfected and after 22 h of transfection, they were changed to serum-free DMEM medium and after 2 hours of starvation, the wells were scribed using a 200 µL pipette. After washing the cells with PBS, DMEM medium containing 1% serum was added to the cells, and cell migration photographs were taken under the microscope at 0, 12, and 24 h, respectively.

### 2.15 Western blotting

The verification of FPR3 protein expression levels was conducted using Western blotting. Initially, the samples underwent treatment with RIPA lysis buffer, and the measurement of protein content was conducted with a BCA test kit (Beyotime, China). Subsequently, an equivalent quantity of protein (20 mg) was loaded into lanes, followed by electrophoresis separation and transfer of the proteins to a PVDF membrane (Merck Millipore, Germany). Primary antibodies were incubated on the membrane after blocking it with a 5% milk solution. The main antibodies used were anti-FPR3 (orb39488, Biorbyt, Cambridge, United Kingdom) and anti-α-tubulin (66,031-1-lg, proteintech, China) diluted at 1:1,000. The incubation was carried out overnight at a temperature of 4°C. After incubation, the secondary antibody was applied at room temperature for an hour. This stage detected the targeted proteins using the enhanced chemiluminescence kit (Servicebio, China).

### 2.16 Statistical analysis

Bioinformatics statistical analyses were conducted using R version 4.1.0, with significance levels set at *P* < 0.05 to determine statistical significance.

## 3 Results

### 3.1 Differential expression and prognostic significance of FPR3 across cancers

**FIGURE 1:**
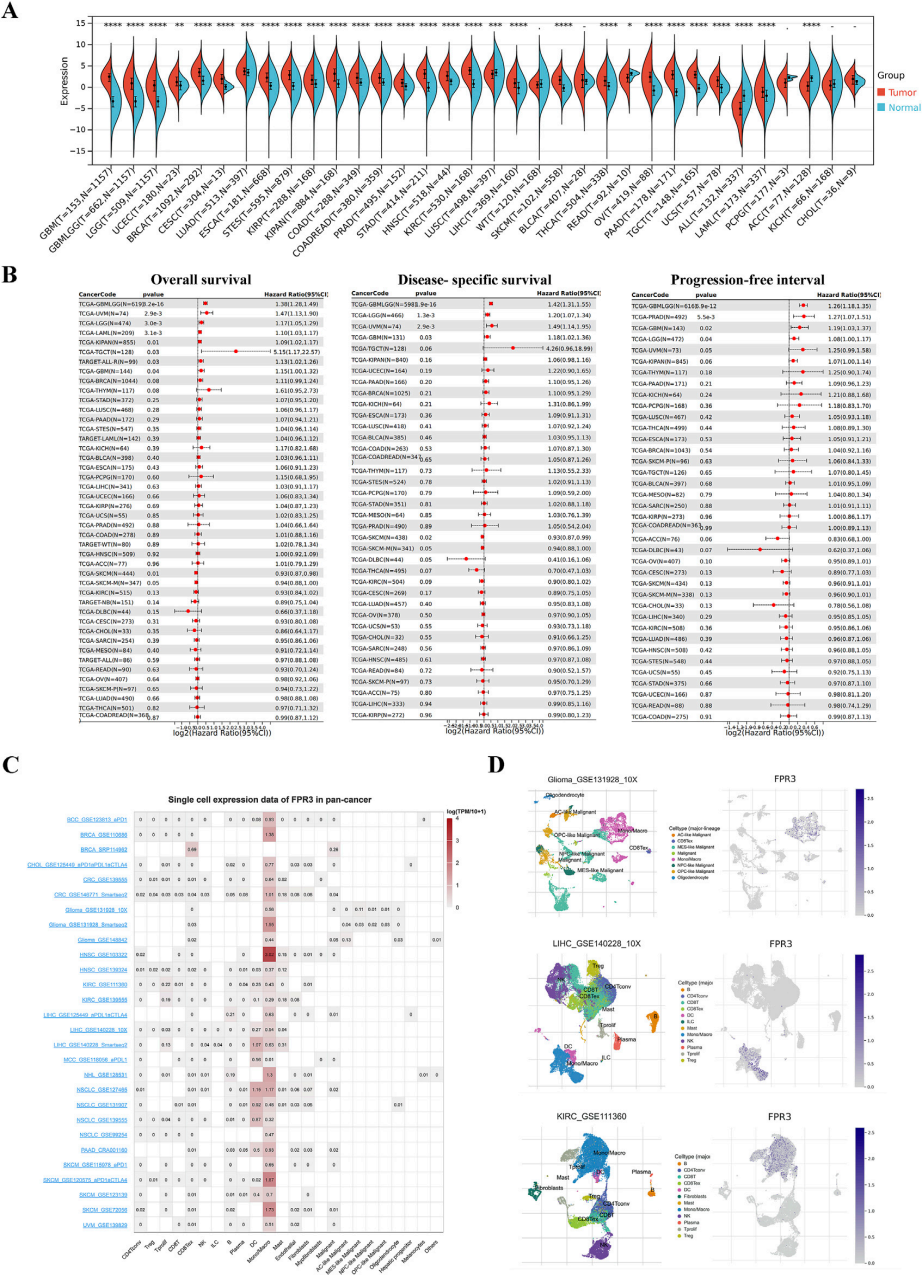
Differential expression and prognostic significance of FPR3 across cancers. **(A)** Differences in FPR3 between normal and tumor tissues, as determined by TCGA and GETx data. **(B)** Forest diagram of FPR3 expression and OS, DSS, and PFI across malignancies. **(C)** Single-cell expression datasets of FPR3. **(D)** Cell type distribution of FPR3 in GSE131928, GSE111360, and GSE140228 datasets.*, *P* < 0.05; **, *P* < 0.01; ***, *P* < 0.001.

FPR3 expression was elevated in the majority of tumor tissues, with a marked decrease in LUSC, ALL, and ACC compared to normal tissues. Noteworthy, no notable variances were observed in WT, BLCA, PCPG, KICH, and CHOL tissues (Figure 1A). We then evaluated the predictive value of FPR3 for OS, PFI, and DSS (Figure 1B). FPR3 expression levels predicted numerous cancer outcomes. Specifically, FPR3 was identified as a protective factor for SKCM but a risk index for GBMLGG, UVM, LGG, LAML, KIPAN, TGCT, and GBM for OS. The upregulation of FPR3 was associated with shorter DSS in GBMLGG, LGG, UVM, and GBM, while SKCM exhibited longer DSS. For PFI, FPR3 expression was a risk factor in GBMLGG, PRAD, GBM, and LGG.

### 3.2 Analysis of FPR3 in single cells

Single-cell sequencing serves as a potent technique for scrutinizing oncogene expression at the individual cell level. Through scRNA sequencing, we were able to identify FPR3 expression in malignant cells as well as immune cells like CD8Tex cells, dendritic cells (DC), and macrophages within BRCA, glioma, GBM, LIHC, NSCLC, and SKCM samples (Figure 1C). Figure 1D displays the expression of FPR3 in the single-cell datasets GSE131928, GSE111360, and GSE140228.

### 3.3 Association between FPR3 expression and immunomodulators, TMB and MSI

In various malignancies such as BLCA, BRCA, COAD, GBM, and LUAD, FPR3 expression correlated positively with immune checkpoint factor expression. Among these immunoinhibitors (Supplementary Figure S1A), FPR3 exhibited the strongest correlations with CSF1R, HAVCR2, PDCD1LG2, and TIGIT. Regarding immunostimulators, FPR3 demonstrated the highest correlations with CD80, CD86, and TNFSF13B (Supplementary Figure S1B). Furthermore, FPR3 was strongly correlated with most MHC molecules (Supplementary Figure S1C). Supplementary Figure S1D shows that FPR3 and TMB are positively linked in UCEC, THYM, SARC, PRAD, OV, LGG, COAD, and BRCA, but negatively correlated in THCA, TGCT, PAAD, LUSC, LIHC, and GBM. UCEC and COAD exhibit a positive correlation between FPR3 expression and MSI, whereas TGCT, STAD, SKCM, OV, LUSC, LUAD, LIHC, LGG, and KIRP exhibit a negative correlation (Supplementary Figure S1E).

### 3.4 Genetic alterations of FPR3 in pan-cancer

Overall, our comprehensive analysis of FPR3 alterations in various cancers utilizing the TCGA dataset and COSMIC database revealed intriguing insights. The FPR3 gene exhibited alterations in 2% of patients across 32 studies and 10,967 samples (Supplementary Figure S2A), with 98 missense, 10 truncating, and 1 fusion mutations identified in amino acids 0–353. Notably, the R137 H/C mutation site was prevalent in UCEC instances (Supplementary Figure S2B). FPR3 genetic mutation in TCGA tumour samples revealed unique patterns. The greatest number of FPR3 alterations (>5%) was observed in patients with UCS, with “Multiple Alterations” being the most prevalent category of alteration (Supplementary Figure S2C). Among the five cancer types, SKCM, UCEC, COADREAD, LIHC, and LUAD, “Mutation” occupied the most common type of FPR3 alterations. In LGG, the FPR3 alteration was primarily characterised by “Deep Deletion”. Supplementary Figure S2D shows Pan-Cancer FPR3 CNV states. Heterozygous Amplification and Heterozygous Deletion are the two main types of CNV in all cancers, The former as the main type of cancer include ACC, UCS, BLCA, KICH, CESC, DLBC, GBM, BRCA and LUSC, while the former as the main type of cancer include SARC, LUAD,TGCT,OV and LGG. The COSMIC database results demonstrated that the major form of FPR3 mutations was missense mutation (35.56%), and the primary SNV was C>T (38.91%) (Supplementary Figure S2E).

### 3.5 FPR3 expression and glioma clinical features

**FIGURE 2:**
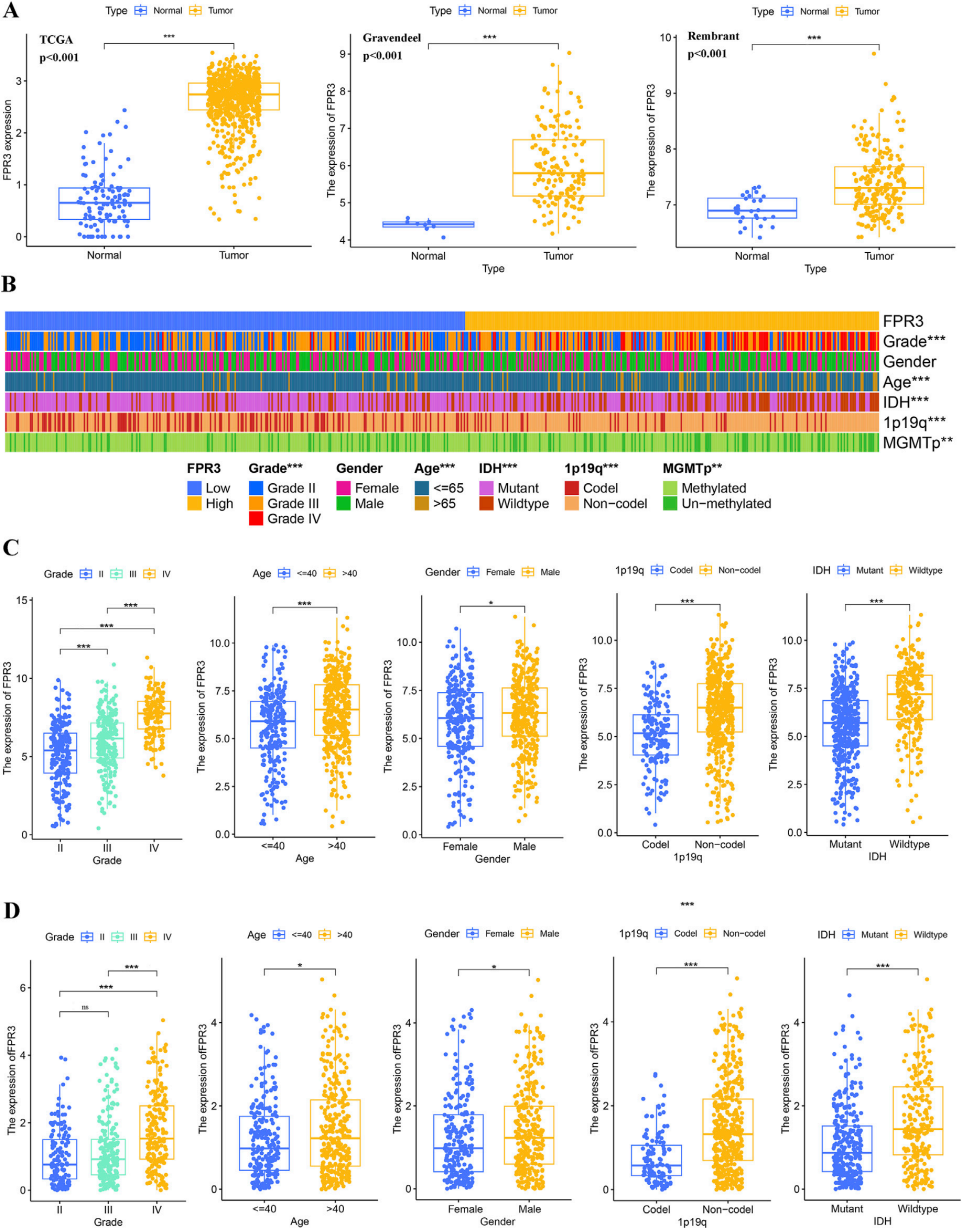
FPR3 expression increases in gliomas. **(A)** An upregulation of FPR3 expression is observed in gliomas that were analyzed using the TCGA + GTEx, Rembrandt, and Gravendeel datasets. **(B–D)** FPR3 expression and associated clinical parameters. *P < 0.05, **P < 0.01, ***P < 0.001.

By integrating GTEx data with TCGA, we performed a differential expression analysis. The investigation demonstrated that glioma tissues exhibited significantly elevated levels of FPR3 expression in comparison to normal tissues (*p* < 0.001). This finding was further corroborated by data from the Rembrandt and Gravendeel datasets (Figure 2A). The cohort with high FPR3 expression displayed a higher incidence of grade III tumors, IDH wild-type status, unmethylated MGMTp, and 1p19q non-codeletion (*p* < 0.01) (Figure 2B). Analysis of the TCGA dataset showed that individuals over the age of 40, with grade III tumors, wild-type IDH status, and 1p19q non-codeletion, had elevated levels of FPR3 expression (*p* < 0.001) (Figure 2C). This discovery was further confirmed by utilizing the CGGA_693 validation dataset (Figure 2D). These outcomes highlight a robust association between elevated FPR3 expression and aggressive features in gliomas.

### 3.6 Prognostic potential of FPR3 in glioma

**FIGURE 3:**
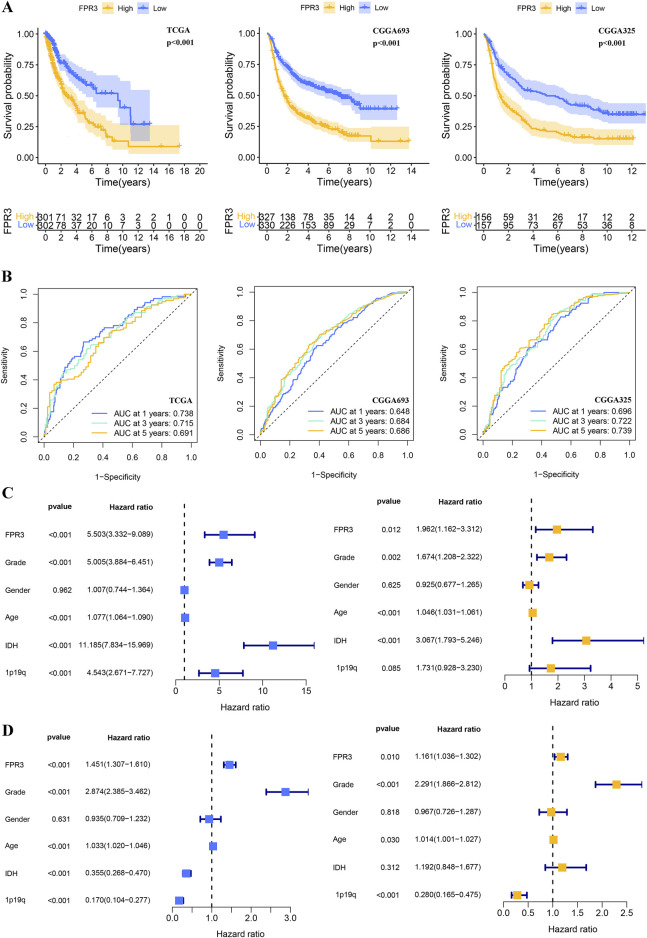
The prognostic value of FPR3 in gliomas. **(A)** For the TCGA-glioma training, CGGA validation datasets, a K-M analysis of OS was performed using FPR3 high-expression versus low-expression in glioma patients. **(B)** Time-ROC analysis demonstrating the prognostic significance of FPR3 in glioma. The results include Cox regression analyses of clinical characteristics and FPR3 expression data: **(C)** results of the TCGA-glioma training database; **(D)** results of the CGGA_325 validation datasets.

In order to assess the prognostic significance of FPR3 in glioma, samples from TCGA and CGGA were categorized into groups based on their FPR3 expression levels relative to the median. The group with elevated FPR3 levels comprised a higher proportion of patients. In the TCGA dataset, elevated FPR3 expression significantly correlated with reduced OS. (*p* < 0.001). Comparable findings were noted in the validation datasets from CGGA (Figure 3A). Additionally, time-ROC curve analysis of FPR3 expression in the TCGA database yielded area under the curve (AUC) values exceeding 0.65, consistent with the findings from the CGGA data (Figure 3B). In glioma patients, increased FPR3 expression may predict poorer outcomes. To identify independent prognostic determinants, Cox regression analysis with one or more variables was conducted. Within the TCGA cohort, FPR3 showed an independent correlation with OS (*p* < 0.05). Additionally, tumor grade, patient age, and IDH status also independently influenced prognosis (Figure 3C). The CGGA_325 validation dataset was used to confirm these findings (Figure 3D). In conclusion, these findings indicate that FPR3 holds promise as a prognostic biomarker for assessing glioma patients.

### 3.7 Building and validating a predictive nomogram

**FIGURE 4:**
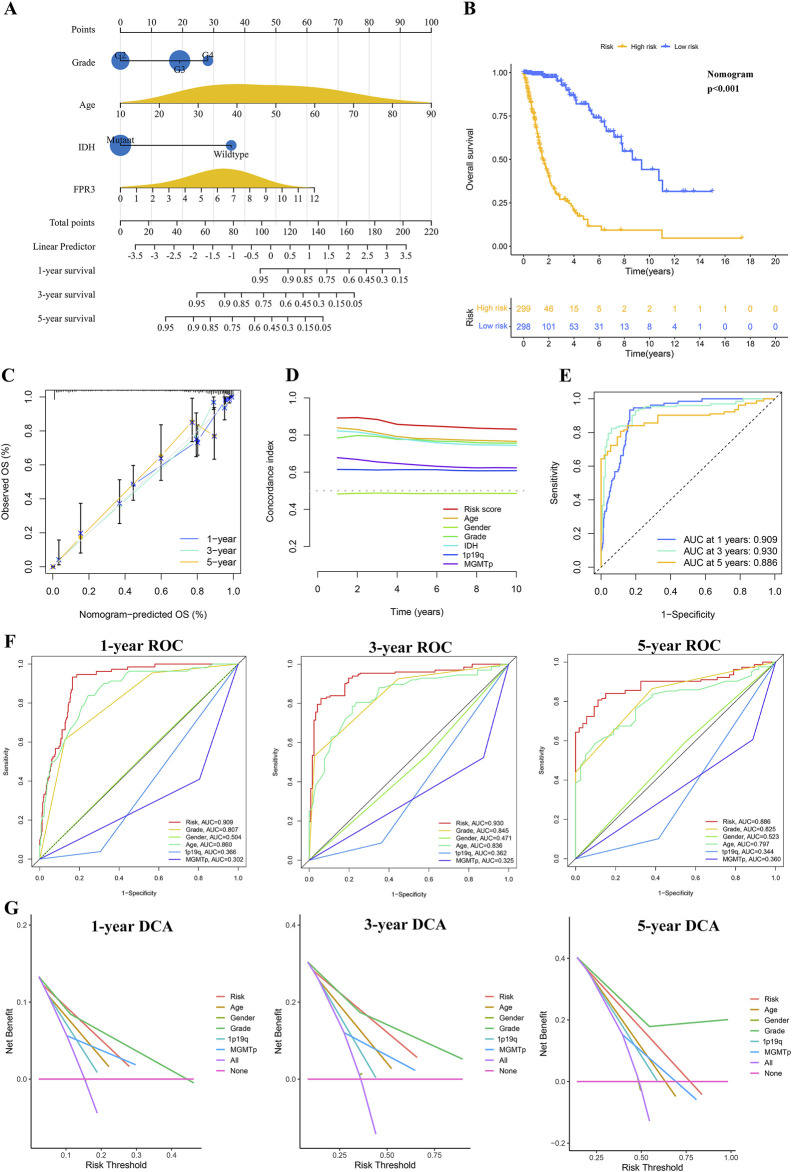
The process of developing and confirming the prognostic nomogram. **(A)** Nomograms were generated to predict the prognosis of glioma patients. **(B)** K-M curve for TCGA glioma datasets based on the nomogram. **(C)** The TCGA datasets’ calibration curves for predicting OS from one to three to 5 years. **(D)** The concordance index (C-index) is generated by the OS nomogram. **(E–F)** Time-ROC analysis demonstrating the nomogram’s prognostic value. **(G)** DCAs for predicting OS using the nomogram.

A nomogram was developed to precisely forecast survival outcomes (Figure 4A). This nomogram incorporated age, grade, IDH status, and FPR3 expression as parameters, which were determined through stepwise Cox multivariate regression analysis. Following this, risk scores were computed utilizing the model. Subsequently, individuals in the training dataset were categorized into two separate groups according to their risk scores. The K-M survival curve demonstrated a notable discriminative capacity of the nomogram. (*p* < 0.001) (Figure 4B). A calibration curve evaluated the agreement between predicted and observed risks (Figure 4C). The calibration curve showed satisfactory alignment between the nomogram-derived and measured OS rates. Furthermore, the model demonstrated exceptional predictive capabilities for OS, as evidenced by the higher C-index and AUC values obtained through time-ROC analysis (Figures 4D–F). These results surpassed those achieved by other clinical and molecular characteristics. Significantly, the DCA curve offered proof endorsing the favorable predictive precision of the nomogram over 1-, 3-, and 5-year periods (Figure 4G).

### 3.8 Conducting functional enrichment analysis on DEGs

**FIGURE 5:**
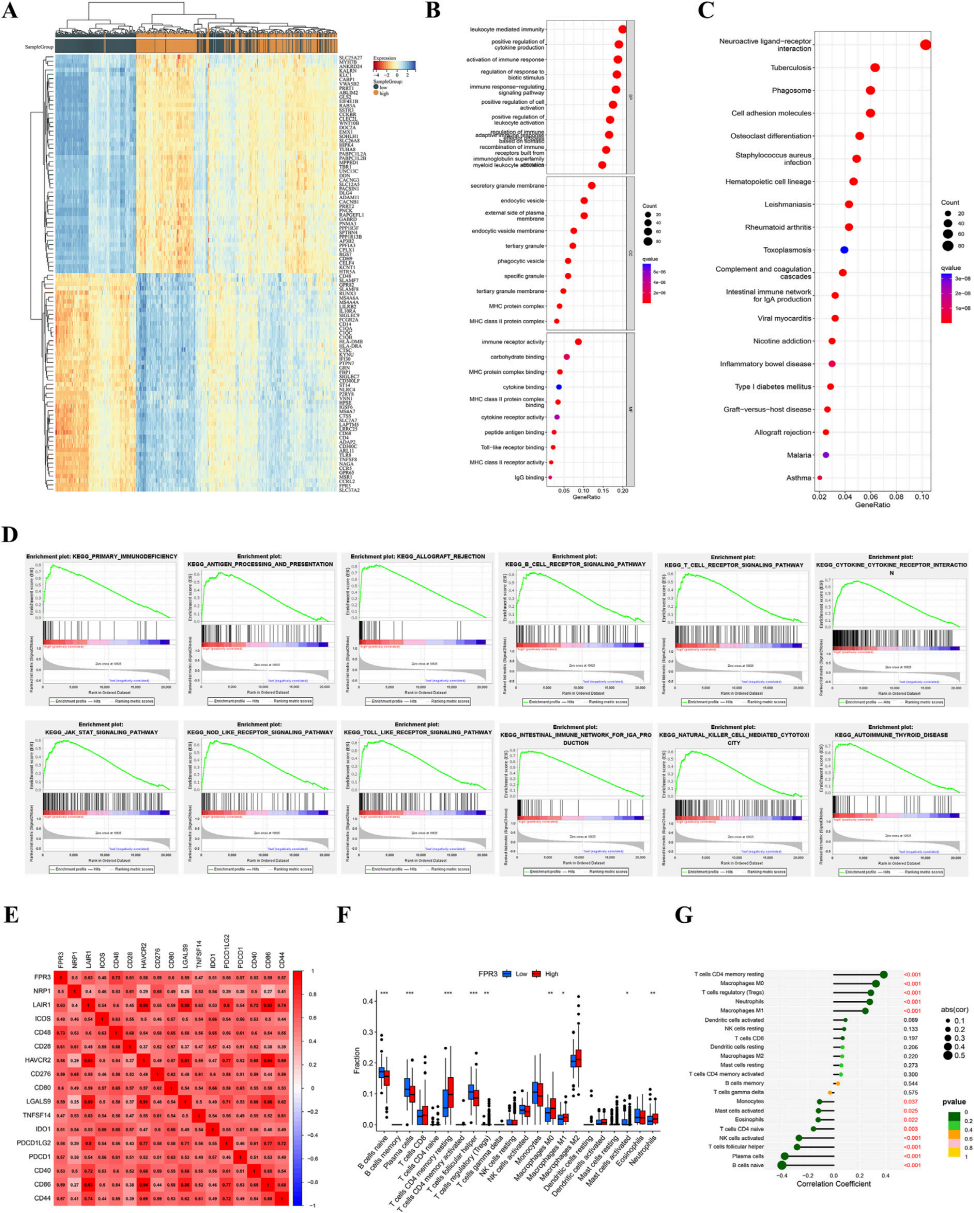
Functional analysis of DEGs in TCGA glioma. **(A)** Heatmap of the TOP 50 filtered DEGs. **(B–C)** GO and KEGG study of FPR3-coexpressed genes in gliomas. **(D)** KEGG signaling pathway analysis. **(E)** FPR3-immune checkpoint molecule connection. **(F–G)** The TCGA dataset reveals varying proportions of 22 subtypes of immune cells.

The Wilcoxon test identified 1,892 differentially expressed genes. A heatmap was created to visually represent the top 50 genes that exhibited significant correlation with FPR3 expression in gliomas (Figure 5A). This analysis revealed 1,112 genes positively associated with FPR3 and 780 genes negatively associated with FPR3. Functional enrichment analysis was performed on genes that positively linked with FPR3, and the results were successfully shown using a bubble chart (Figure 5B). The GO term annotations revealed that the genes are implicated in various biological processes, including leukocyte-mediated immunity and immune response-regulating signaling pathways. KEGG study linked these genes to neuroactive ligand-receptor interaction, phagosome formation, and cell adhesion molecules (Figure 5C). GSEA techniques were employed to identify signaling pathways associated with FPR3 in gliomas. Increase in FPR3 expression was identified to be pivotal in various processes, including the signaling pathways of B-cell receptors, T-cell receptors, and Toll-like receptors (Figure 5D).

### 3.9 FPR3 and immune cell infiltration in glioma

Correlations exist between the FPR3 gene expression and 28 different classifications of TILs. Supplementary Figure S3A illustrates a significant association between FPR3 expression levels and TILs across various human cancers, with a particular emphasis on gliomas. Additionally, a TISIDB analysis of FPR3 levels in LGG immune subgroups revealed that the C3 subtype exhibited the highest expression levels, while the C5 subtype showed reduced expression (Supplementary Figure S3B). The ESTIMATE algorithm also compared glioma immune infiltration patterns to FPR3 expression. The findings suggested that groups with high expression levels exhibited elevated immunological, stromal, and ESTIMATE scores in contrast to those with low expression levels (Supplementary Figure S3C). FPR3 was examined in connection to immune checkpoint target genes. The FPR3 protein positively correlated with CD48, CD28, CD80, CD86, and PDCD1 (Figure 5E). Moreover, FPR3 expression exhibited connections with macrophage abundance, Tem_CD8 abundance, Myeloid-derived suppressor cell abundance, mast cell abundance and regulatory T cell abundance in GBM (r > 0.65, *p* < 0.05) (Supplementary Figure S3D). Similarly, this trend was observed in LGG. Figures 5F, G shows intergroup differences in 22 immune cell subpopulations. In the high FPR3 expression group, there were elevated levels of resting memory CD4 T cells, regulatory T cells, M0 and M1 macrophages, and neutrophils, whereas the low FPR3 expression group displayed increased proportions of naive B cells, plasma cells, and follicular helper T cells. We also examined FPR3 expression and gene markers associated with distinct TILs (Table 1). In general, the expression of FPR3 was significantly associated with gene markers for B cells, T cells, and M1 and M2 macrophages.

**TABLE 1:** Correlation between FPR3 expression and immune cell gene markers.

Immune cell types	Gene markers	GBM	P	LGG	P
		Cor		Cor	
B cells	CD2	0.67	***	0.58	***
	CD74	0.76	***	0.63	***
	CD27	0.65	***	0.41	***
Plasma cells	MAST1	−0.43	***	−0.3	***
	MANEA	0.36	***	0.29	***
T cells	CD3E	0.64	***	0.58	***
	CD3D	0.63	***	0.56	***
CD4+T cells	CD4	0.81	***	0.76	***
CD8^+^ T cell	CD8A	0.43	***	0.44	***
Tfh	CD84	0.79	***	0.64	***
	IL6R	0.57	***	0.55	***
M1 Macrophage	CD80	0.67	***	0.72	***
	IRF5	0.74	***	0.54	***
	CD64	0.68	***	0.49	***
M2 Macrophage	CD163	0.67	***	0.67	***
	CD206	0.68	***	0.41	***
	VSIG4	0.69	***	0.67	***
	MS4A4A	0.75	***	0.79	***
Neutrophils	CCR1	0.78	***	0.76	***
	CCR7	0.41	***	0.42	***
	SLC1A5	0.53	***	0.48	***
	CXCR2	0.47	***	0.44	***
	ITGAM	0.8	***	0.59	***
Dendritic cell	CD8A	0.43	***	0.44	***
	CD141	0.65	***	0.45	***
	NRP1	0.62	***	0.46	***
	THBD	0.65	***	0.45	***
	ITGAX	0.54	***	0.45	***
	HLA-DPB1	0.79	***	0.62	***
	HLA-DQB1	0.68	***	0.46	***
	HLA-DRA	0.77	***	0.64	***
	HLA-DPA1	0.72	***	0.6	***

### 3.10 FPR3 expression and drug susceptibility

We investigated the link between FPR3 expression and drug sensitivities using common medicines or chemicals. Through a comparative assessment of IC50 values in two different groups, we found that the cohort with elevated FPR3 expression had significantly lower IC50 values for Temozolomide, AMG-319, Bortezomib, Cediranib, Dasatinib, Entospletinib, Savolitinib, and 5-Fluorouracil compared to the group with lower expression levels. In contrast, the high-expression group demonstrated higher IC50 values for SB505124 and Vorinostat than the low-expression group (Supplementary Figure S3E).

### 3.11 The expression of FPR3 in glioma was confirmed by experimental verification

**FIGURE 6:**
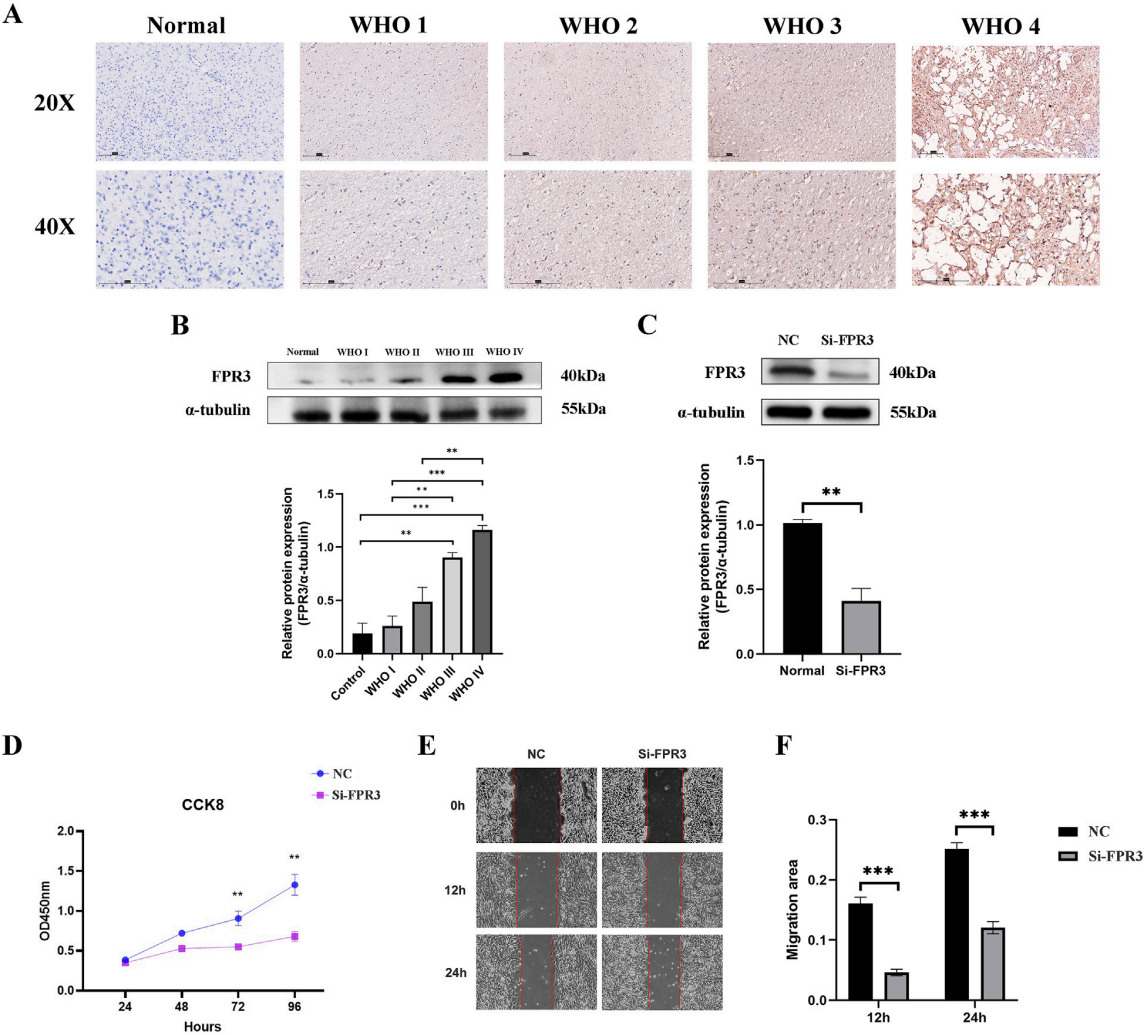
Experimental verification of FPR3. **(A)** The difference of FPR3 expression between normal tissues and different grades of glioma tissues was demonstrated by immunohistochemistry. The scale bar measures 150 mm. **(B)** The distinction in FPR3 expression between normal tissues and various classifications of glioma tissues was demonstrated by Western blot. **(C)** Western blot analysis was used to evaluate the efficacy of siRNA specifically engineered to target FPR3 after transfection in U251 cells. **(D)** A CCK-8 test was used to assess the effect of FPR3 knockdown on cellular proliferation. **(E, F)** The findings from wound healing trials indicate that the suppression of FPR3 may successfully restrict the migration of U251 cells.

The levels of FPR3 protein in glioma were verified using immunohistochemistry and Western blot. The results consistently indicated an upregulation of FPR3 protein expression in glioma specimens compared to the paired adjacent specimens (Figures 6A, B).

### 3.12 The knockdown of FPR3 suppresses the proliferation and migration of glioma cell

To investigate the biological role of FPR3 in GBM, its expression was significantly reduced in U251 cells transfected with siRNA-FPR3 (Figure 6C). The CCK-8 assay revealed that FPR3 suppression hindered GBM cell proliferation compared to the NC cells (Figure 6D). Additionally, the wound healing assay further demonstrated that the migratory capacity of GBM cells was significantly reduced in the FPR3 knockdown cells (Figures 6E, F). These findings suggest that dysregulated FPR3 expression may affect both the proliferation and migration of GBM cells.

## 4 Discussion

FPR3 has potential as both a diagnostic marker for specific types of cancer (Qi et al., 2020). A recent study demonstrated that FPR3 inhibits the AKT/mTORC1 signaling pathway by modulating cellular calcium ion fluxes, thereby impeding the progression of gastric cancer (Wang et al., 2024). However, the role of FPR3 in other malignancies remains uncertain. Based on our findings, FPR3 has the strongest association with glioma. Therefore, we further analyzed FPR3 expression in glioma, its prognostic significance concerning immune infiltration, and performed functional analysis. Our pan-cancer analysis revealed that FPR3 is upregulated in 25 tumors, including GBM and LGG. In individuals with LGG, GBM, and UVM, elevated levels of FPR3 expression were linked to a worse prognosis. FPR3 was previously identified as one of six immune-related genes in a study analyzing the expression and functionality of low-grade gliomas (Tan et al., 2020). The prognostic analysis, immune infiltration, and *in vitro* experimental validation from the previous research were not confirmed. Our current work aims to provide supplementary validation.

This research investigated FPR3 as a potential indicator of immunotherapy effectiveness. Numerous cancer types demonstrated high correlations between FPR3 expression and both immune checkpoint molecules and TMB levels. According to a recent study (Newman et al., 2020), ICB therapy was more effective in patients with higher TMB levels. In eight cancers, FPR3 expression was positively linked with TMB, with THYM, OV, LGG, and COAD showing the most significant associations. Additionally, increasing evidence suggests that gastric cancer patients with MSI status have better survival rates (Cristescu et al., 2015; Miceli et al., 2019). The molecular characteristics of MSI malignancies may render them more responsive to cancer immunotherapy (Di Bartolomeo et al., 2020). Multiple cancers showed a substantial connection between FPR3 expression and MSI, confirming its immunotherapy potential.

Gliomas are the main reason for death related to primary brain tumors and account for approximately 80% of aggressive brain malignancies (Chen et al., 2017). Traditional therapies encounter intrinsic limitations, leading to a decreased overall survival rate (Goodenberger and Jenkins, 2012). To address the limitations of current treatment modalities, there has been a surge in research exploring cancer immunotherapy as a potential therapeutic approach for gliomas. Immunotherapy has the potential to achieve sustained tumor remission by modulating the immune system while minimizing adverse effects (Sanmamed and Chen, 2018). Recent research has provided insights into the potential of immunotherapy to elicit anti-tumor responses in the brain, presenting a promising opportunity for developing therapeutic approaches against malignant gliomas (Zhang et al., 2021). Consequently, it has become crucial in clinical practice to identify new biomarkers.

This analysis utilized publicly available databases, including TCGA, CGGA, Rembrandt, and Gravendeel. Examination of these databases revealed that FPR3 overexpression significantly correlated with multiple clinical indicators. This study suggests that FPR3 may serve as a valuable molecular marker for cerebral gliomas. FPR3 expression, along with age, grade, and IDH status, were integrated to develop a nomogram for outcome prediction. This nomogram aims to enhance the incorporation of FPR3 into clinical treatments. Our nomogram performed well in TCGA training and CGGA validation statistical assessments. Our research successfully identified and confirmed the significance of FPR3 expression as a valuable and independent predictive indicator for glioma. Since FPR3 has a potential cancer-promoting effect in various cancers, including GBM, and the oncogenic role of FPR3 in GBM were then tested *in vitro* in GBM cell lines. FPR3 knockdown remarkably impaired the proliferation and migration abilities of U251 cells.

To obtain a more comprehensive understanding of FPR3’s involvement in gliomas, we conducted a examination of DEGs using GO and KEGG enrichment analyses. These analyses demonstrated that FPR3 is linked to immune-related activities and signaling pathways. Furthermore, GSEA successfully identified multiple gene sets linked to tumors, including hallmark gene sets related to T-cell receptor signaling pathways. T cells may directly sense danger signals utilizing Toll-Like Receptor expression, according to previous findings (Choi et al., 2007). The goal of cancer immunotherapy is to provoke and sustain a robust immune response against cancerous growths by activating T cells (Chen and Mellman, 2013; Spitzer et al., 2017). The signaling mechanisms of T cell receptors are vital to the immune regulatory processes in glioma (Claes et al., 2012). Recent advancements in brain tumor research have shown the promising efficacy of immunotherapy as a treatment for glioma, primarily due to its ability to attract tumor-infiltrating T lymphocytes, potentially leading to tumor cell eradication (Bruno et al., 2017).

Recent research has indicated that TILs can independently predict cancer onset (Azimi et al., 2012). In glioma, FPR3 expression shows a positive correlation with immune infiltration, particularly involving immune cells linked to anti-tumor activity, such as regulatory T cells, central memory CD8 T cells, and macrophages, according to an analysis of cancer classifications in the database. Prior research has demonstrated that gene expression patterns in tumour tissues are influenced by the immune microenvironment, and the prognosis is contingent upon the degree of infiltration facilitated by stromal and immune cells (Winslow et al., 2016). In our investigation, immunological, stromal, and ESTIMATE scores were significantly elevated in correlation with elevated FPR3 expression.

Immune cell regulation, whether intrinsic or by intercellular contact with cancer cells, is critical to the genesis of neoplastic diseases (Kamran et al., 2018). In the process of immunomodulation, checkpoint molecules play an essential part, and recent research indicates that blocking these molecules may hold substantial promise for the treatment of gliomas using immune therapies (Saha et al., 2017). FPR3 correlated positively with immune checkpoint genes, particularly PD-1 and CD48, suggesting its potential regulation of diverse immunological checkpoints in gliomas. Anti-PD-1 treatment is thought to fight cancer by stopping cell surface-expressed PDL1 from inhibiting PD1+ antitumor T lymphocytes (Dong et al., 2002). Upregulation of PD-L1 was predominantly observed in highly aggressive phenotype glioma cells, which subsequently migrate and activate the PI3K/Akt/actin pathway (Chen et al., 2019). Tumor immunotherapy, particularly the modulation of immune checkpoints by suppressing FPR3 expression, has garnered significant attention in our research. Additionally, we explored the potential use of FPR3 as a viable strategy to enhance immune checkpoint blockade therapy, aiming to optimize treatment efficacy while mitigating adverse effects. Additional research is required to completely understand and elucidate the processes. This current investigation presents innovative perspectives that can contribute to future scholarly inquiries into the molecular mechanisms under consideration.

The ongoing investigation has several limitations. Firstly, there was a significant reliance on internet-based public databases and computational methodologies, due to the scope and limitations of our current study, we are unable to perform sequencing for FPR3 detection at this time. We believe that future research incorporating clinical outcomes will complement our findings and provide a more complete picture. Furthermore, our experimental validation was limited to *in vitro* studies, additional vivo investigations are necessary. This research investigated the levels of FPR3 and the presence of immune cells infiltrating glioma tissues in patients. However, accurately determining the specific role of FPR3 in regulating the microenvironment in gliomas is challenging. Therefore, understanding the unique characteristics of the unidentified ligand associated with FPR3 and thoroughly investigating the signaling cascades activated in both neoplastic and immune cells are crucial.

## 5 Conclusion

Our research has demonstrated a notable elevation in FPR3 levels across multiple tumor types, closely linked to unfavorable patient outcomes. Furthermore, FPR3 presence is related to the infiltration of diverse immune cells, possibly affecting the glioma immune microenvironment. These findings indicate that FPR3 could be a valuable prognostic marker and an essential target for immunotherapy in glioma treatment. Consequently, this discovery bears considerable clinical relevance.

## Data Availability

Publicly available datasets were analyzed in this study. This data can be found here: https://portal.gdc.cancer.gov/repository.
