# Supplementary figures and images for: Pan-cancer analysis and experimental validation of FPR3 as a prognostic and immune infiltration-related biomarker for glioma
Source: Front Genet. 2024 Oct 9;15:1466617. doi: 10.3389/fgene.2024.1466617 (PMC11496095; doi:10.3389/fgene.2024.1466617)

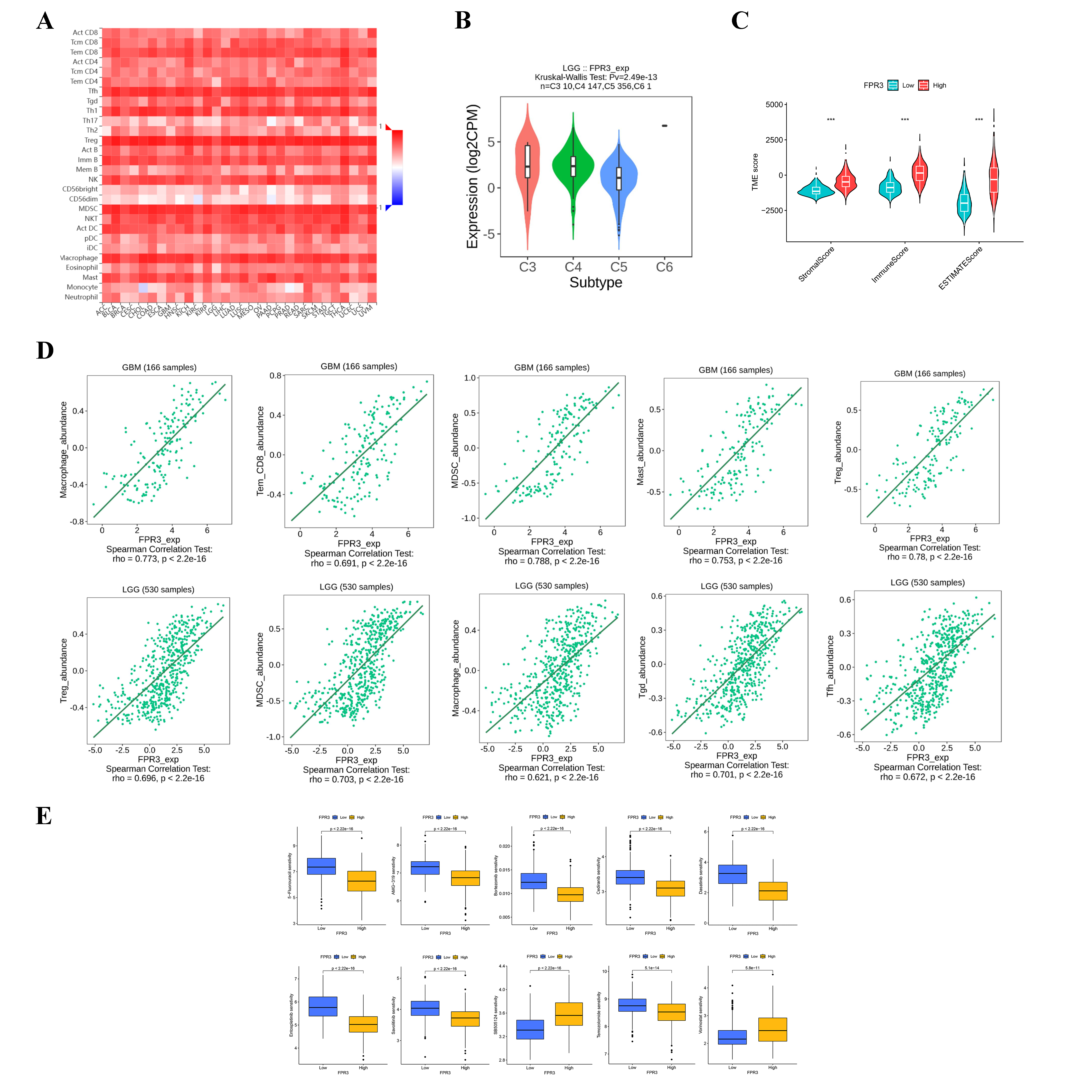

Supplement: Supplementary file 1 [file Image3.TIF]

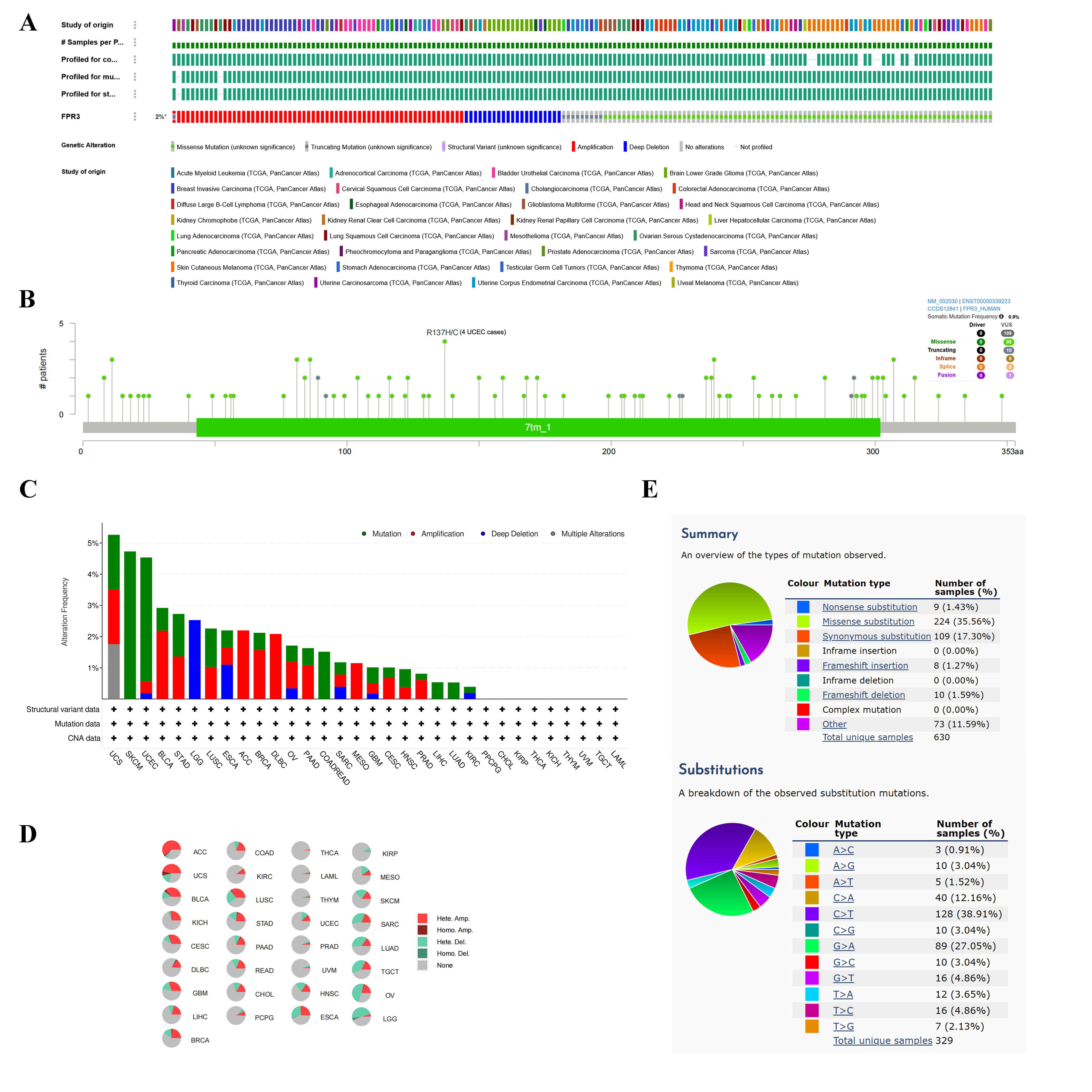

Supplement: Supplementary file 2 [file Image2.TIF]

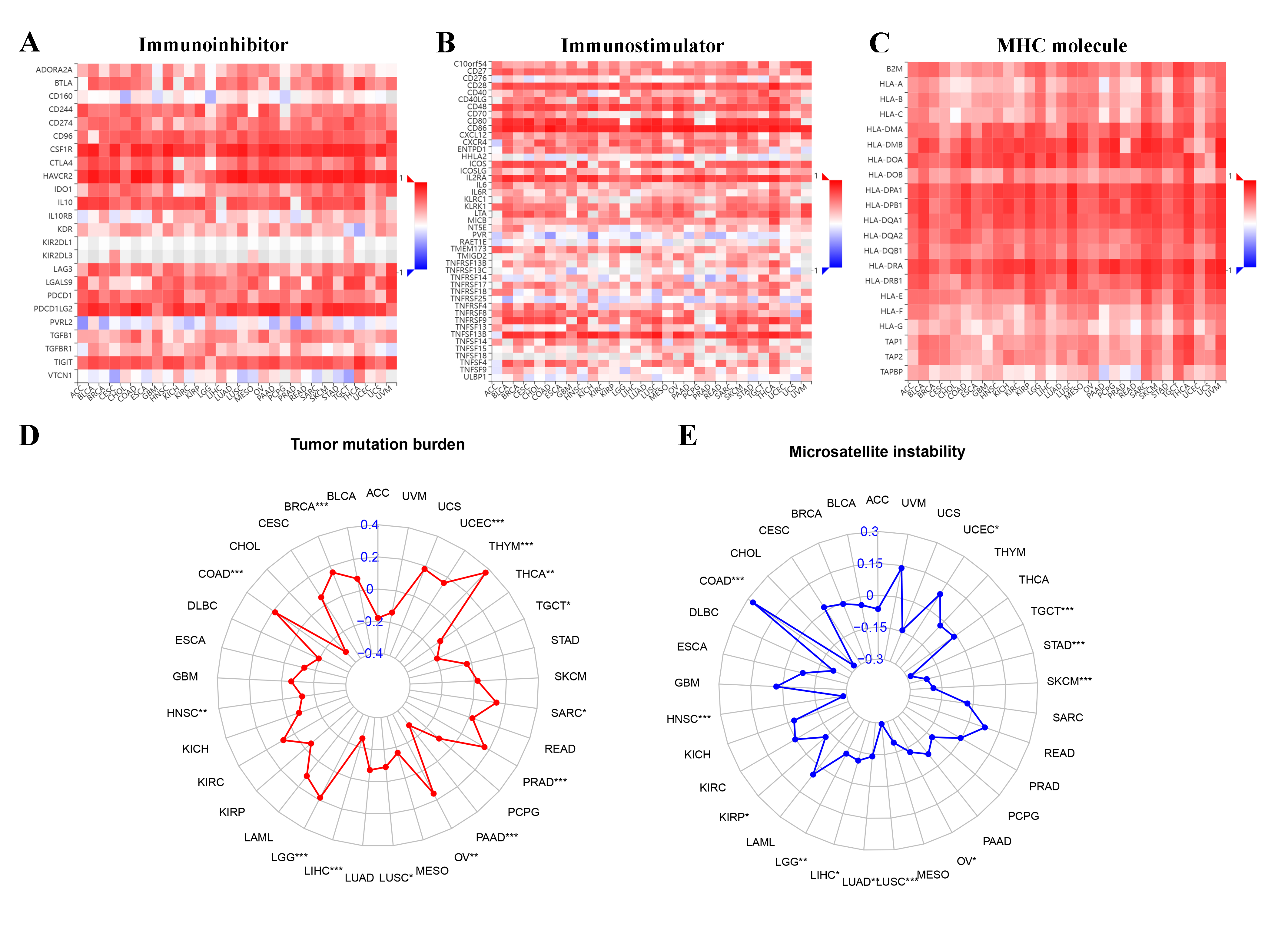

Supplement: Supplementary file 3 [file Image1.TIF]
